# PW02-037 - The Eurofever cohort of 136 patients with CAPS

**DOI:** 10.1186/1546-0096-11-S1-A178

**Published:** 2013-11-08

**Authors:** R Levy, L Gerard, J Kuemmerle-Deschner, H Lachmann, I Koné-Paut, L Cantarini, P Woo, A Naselli, B Bader-Meunier, F De Benedetti, SM Al-Mayouf, S Ozen, M Hofer, J Frenkel, C Modesto, I Nikishina, A Martini, N Ruperto, B Neven, M Gattorno

**Affiliations:** 1 for PRINTO and Eurofever Project, Paris, France; 2 for PRINTO and Eurofever Project, Tübingen, Germany; 3 for PRINTO and Eurofever Project, London, UK; 4 for PRINTO and Eurofever Project, Siena, Italy; 5 for PRINTO and Eurofever Project, Genova, Italy; 6 for PRINTO and Eurofever Project, Roma, Italy; 7 for PRINTO and Eurofever Project, Riyadh, Saudi Arabia; 8 for PRINTO and Eurofever Project, Ankara, Turkey; 9 for PRINTO and Eurofever Project, Lausanne, Switzerland; 10 for PRINTO and Eurofever Project, Utrecht, Netherlands; 11 for PRINTO and Eurofever Project, Barcelona, Spain; 12 for PRINTO and Eurofever Project, Moscow, Russian Federation

## Introduction

Cryopyrin associated periodic syndromes (CAPS) is a spectrum of autoinflammatory syndromes including familial cold autoinflammatory syndrome, Muckle Wells syndrome and CINCA (chronic infantile neurologic articular syndrome). All phenotypes are associated with gain of function mutation in *NLRP3* encoding cryopyrin, a protein involved in inflammasome and IL-1b processing.

## Objectives

We describe clinical features in a large cohort of patients and test the hypothesis of a génotype/phénotype correlation.

## Methods

Members of PRINTO were invited to report their patients to the Eurofever registry via a web-based questionnaire. Experts in CAPS validated diagnosis in 136 patients. Mean age of the cohort at inclusion was 26 years.

## Results

Mean age at disease onset was 5 years. Skin rash, articular involvement and fever were the most prevalent features, respectively in 132, 117 and 108 patients. 6 patients suffered from severe articular involvement defined as flexion contractures, patella overgrowth or bone complications. Neurological involvement was noticed in 55 patients and characterized by morning headaches (n=39), aseptic meningitis (n=23), papilledema (n=29), Severe neurological involvement (hydrocephalus, mental retardation, seizures) was reported in 16 patients. Ophthalmological involvement was observed in 97 patients suffering from conjunctivitis (n=87), uveitis (n=7), or papilledema. 56 patients presented neurosensorial hearing loss. 78 had a chronic course, while 58 experienced only acute episodes. 76 cases were familial, 54 sporadic. NLRP3 sequencing had been performed in all patients; heterozygous germline mutation was reported in 133 patients. 7 mutations were recurrent and found in 106 patients: R260W (n= 36), T348M (n= 20), A439V (n= 14), V198M (n= 13), E311K (n= 9), Q703K (n= 9), D303N (n= 5), 27 patients carried a non recurrent mutation; Statistically significant associations between genotype and phenotype were found: patients with non recurrent mutations had the most severe phenotype (youngest age of onset, more frequent neurological involvement, including severe ones and severe arthropathies). Patients carrying R260W mutation were associated with acute pattern, cold triggering and onset > 6 months. T348M group was associated with onset < 6 months, a chronic course and frequent hearing loss. V198M, E311K and A439V were less frequently associated with neurological involvement.

## Conclusion

This retrospective large survey of CAPS patients allowed to better define genotype-phenotype correlation. Patients with non-recurrent NLRP3 mutations were identified at risk of severe disease-related complications. Early effective treatment with anti-IL1b drugs and close monitoring should be recommended in these patients.

## Competing interests

None declared

